# A Living Child, 39 Days after Quickening—147 Days after Conception

**Published:** 1844-09-07

**Authors:** 


A LIVING CHILD, 39 DAYS AFTER QUICKENING—147 DAYS AFTER CONCEPTION.

This curious case happened in the practice of Mr. C. Smythe, of Castle Douglass. A female, in her second pregnancy, and in the 147th day of utero-gestation, had a severe flooding, with rupture of the membranes. Labour did not take place till the next night, when a very small but well-formed fœtus was expelled, giving no other indication of life than a very feeble action of the heart, and a strong pulsation in the cord. By proper means, however, it was resuscitated, and cried as strongly as a child born at the full period of pregnancy. It weighed less than two pounds, and measured exactly twelve inches. It swallowed some nourishment, but died at twelve hours and a-half after birth. The Membrana Pupillaris was entire—the testicles had not descended—the head well covered with hair. Some ecchymosis appeared on the back and on the hands before death. From peculiar circumstances, it was evident that the mother of the infant was perfectly correct in respect to dates.

There was clearly nothing in the organisation of this child to prevent its growing to the age of maturity. We believe that there is a case recorded in the Edinburgh Medical and Surgical Journal, where a premature fœtus of eighteen weeks was preserved by great care. In the present case the period of utero-gestation extended to 21 weeks.—*Med. Chir Rev.*

